# Discussion session: biomarker trials

**DOI:** 10.1186/1745-6215-16-S2-I2

**Published:** 2015-11-16

**Authors:** Various speakers

## 

Various speakers In a combined presentation and discussion session, representatives from academia and industry discuss the role of Biomarker trial design and implementation, to improve health by improving trials.

